# Two NADPH: Protochlorophyllide Oxidoreductase (POR) Isoforms Play Distinct Roles in Environmental Adaptation in Rice

**DOI:** 10.1186/s12284-016-0141-2

**Published:** 2017-01-11

**Authors:** Choon-Tak Kwon, Suk-Hwan Kim, Giha Song, Dami Kim, Nam-Chon Paek

**Affiliations:** 1 Department of Plant Science, Plant Genomics and Breeding Institute, Research Institute of Agriculture and Life Sciences, Seoul National University, Seoul, 08826 Republic of Korea; 2 Crop Biotechnology Institute, Institutes of Green Bio Science and Technology, Seoul National University, Pyeongchang, 25354 Republic of Korea

**Keywords:** Rice, *Faded green leaf*, *OsPORA*, *OsPORB*, Chlorophyll synthesis

## Abstract

**Background:**

NADPH: protochlorophyllide oxidoreductase (POR) is an essential enzyme that catalyzes the photoreduction of protochlorophyllide to chlorophyllide, which is ultimately converted to chlorophyll in developing leaves. Rice has two POR isoforms, OsPORA and OsPORB. *OsPORA* is expressed in the dark during early leaf development; *OsPORB* is expressed throughout leaf development regardless of light conditions. The *faded green leaf* (*fgl*) is a loss-of-function *osporB* mutant that displays necrotic lesions and variegation in the leaves due to destabilized grana thylakoids, and has increased numbers of plastoglobules in the chloroplasts. To investigate whether the function of OsPORA can complement that of OsPORB, we constitutively overexpressed *OsPORA* in *fgl* mutant.

**Results:**

In the *35S:OsPORA/fgl* (termed OPAO) transgenic plants, the necrotic lesions of the mutant disappeared and the levels of photosynthetic pigments and proteins, as well as plastid structure, were recovered in developing leaves under natural long days in the paddy field and under short days in an artificially controlled growth room. Under constant light conditions, however, total chlorophyll and carotenoid levels in the developing leaves of OPAO plants were lower than those of wild type. Moreover, the OPAO plants exhibited mild defects in mature leaves beginning at the early reproductive stage in the paddy field.

**Conclusions:**

The physiological function of OsPORB in response to constant light or during reproductive growth cannot be completely replaced by constitutive activity of OsPORA, although the biochemical functions of OsPORA and OsPORB are redundant. Therefore, we suggest that the two OsPORs have differentiated over the course of evolution, playing distinct roles in the adaptation of rice to the environment.

**Electronic supplementary material:**

The online version of this article (doi:10.1186/s12284-016-0141-2) contains supplementary material, which is available to authorized users.

## Background

In angiosperms, chlorophyll (Chl) absorbs light and imparts its energy to other components of the electron transport chain during photosynthesis (Grossman et al. 1995; Barber et al. 2000). Chl is an essential compound in higher plants. However, the intermediate compounds in Chl synthesis can bind to oxygen molecules, leading to the production of reactive oxygen species (ROS) including singlet oxygen radicals (op den Camp et al. 2003). The accumulation of ROS accelerates cellular signaling pathways or oxidizes cellular elements, leading to photo-oxidative damage and cell death (Kim et al. 2008). Therefore, Chl synthesis must be precisely controlled during chloroplast development (Sakuraba et al. 2013). During Chl synthesis, NADPH: protochlorophyllide oxidoreductases (PORs) catalyze the light-dependent conversion of protochlorophyllide (Pchlide) to chlorophyllide (Chlide), a critical intermediate step in the process (Virgin et al. 1963; Henningsen 1970; Griffiths 1978; Masuda and Takamiya 2004).

In angiosperms, during skotomorphogenesis in the dark, PORs combine with NADPH and Pchlide to produce a ternary complex that makes up the main protein component of the prolamellar body (PLB) in the etioplast (Schoefs and Franck 2003; Paddock et al. 2012). The PLB, which has a lattice-like structure, also contains Chl precursors, carotenoids, and lipids (Rosinski and Rosen 1972; Selstam and Sandelius 1984). The NADPH: POR: Pchlide ternary complex reduces the D-ring in Pchlide to produce Chlide, which is esterified and modified to create Chl *a* and Chl *b* in a light-dependent manner (Apel et al. 1980; Lebedev and Timko 1998; Heyes and Hunter 2005). Through the process of light-induced greening, the PLB in the etioplast collapses and the grana thylakoids emerge, resulting in the formation of chloroplasts (Virgin et al. 1963; Henningsen 1970). This photomorphogenesis process in higher plants begins with the visible accumulation of Chl, which is a consequence of the functions of the NADPH: POR: Pchlide ternary complex (Solymosi et al. 2007). At the same time, the transition from etioplasts containing PLBs to chloroplasts with mature thylakoids is closely associated with the role of POR in Chl synthesis (Solymosi et al. 2007). Thus, POR is ultimately involved in the formation of thylakoid membranes in higher plants (Forreiter et al. 1991).

Many gymnosperms, algae, and cyanobacteria can synthesize photosynthetically competent chloroplasts, even in the dark, because they contain two different enzymes, light-dependent POR (LPOR) and dark-operative POR (DPOR) (Forreiter and Apel 1993; Shui et al. 2009); LPOR requires light for its function, but DPOR can function in the absence of light. By contrast, angiosperms possess only LPORs; the functional deficiency of DPOR in higher plants may be related to the adaptation of specific POR regulatory mechanisms (Masuda and Takamiya 2004; Paddock et al. 2010). In addition, phylogenic analysis suggests that gene duplication might have resulted in the formation of POR families (Masuda and Takamiya 2004).

The roles of PORs have been well-studied in angiosperms, including *Arabidopsis thaliana* (Armstrong et al. 1995; Benli et al. 1991; Oosawa et al. 2000), *Nicotiana tabacum* (Zavaleta-Mancera et al. 1999; Masuda et al. 2002), *Zea mays* (Millerd and McWilliam 1968; Hopkins 1982; Hopkins and Elfman 1984), *Avena sativa* (Darrah et al. 1990), *Hordeum vulgare* (Schulz et al. 1989; Holtorf et al. 1995), *Triticum aestivum* (Teakle and Griffiths 1993), *Cucumis sativus* (Yoshida et al. 1995; Fusada et al. 2000), *Amaranthus tricolor* (Iwamoto et al. 2001), *Brassica oleracea* (Solymosi et al. 2004), and *Pisum sativum* (Spano et al. 1992). Three *POR* genes (*AtPORA*, *AtPORB*, and *AtPORC*) have been identified in Arabidopsis (Armstrong et al. 1995; Oosawa et al. 2000). Whereas barley and tobacco have two isoforms of *POR* (Holtorf et al. 1995; Masuda et al. 2002), cucumber and pea possess only a single *POR* (Spano et al. 1992; Fusada et al. 2000).

The biological functions of PORs in barley and Arabidopsis are well known, as these plants are representative monocots and dicots, respectively. The two POR isoforms in barley are distinct, although the enzymatic activities of HvPORA and HvPORB in vitro are similar (Holtorf et al. 1995). For example, *HvPORA* is mainly expressed in etiolated seedlings and is downregulated by illumination, but *HvPORB* is constitutively expressed during leaf development, regardless of light conditions (Holtorf et al. 1995). In Arabidopsis, the functions and expression patterns of *AtPORA* and *AtPORB* are analogous to those of the two *PORs* in barley (Armstrong et al. 1995; Runge et al. 1996). However, *AtPORC* is upregulated in the light and downregulated in the dark (Oosawa et al. 2000; Su et al. 2001). Moreover, *AtPORC* expression strongly increases under high light conditions, indicating that AtPORC activity is crucial for protecting Chl from breakdown under excessive light during plant development (Masuda et al. 2003).

Rice has two POR isoforms, *OsPORA* and *OsPORB*. A mutation in *OsPORB* (*osporB*) was first identified in a study of the *faded green leaf* (*fgl*) mutant, which displays necrotic lesions and variegation during leaf development (Sakuraba et al. 2013). The defects in *fgl* mutant are strongly associated with the reduced expression of *OsPORA* and the lack of OsPORB activity in developing leaves (Sakuraba et al. 2013). Also, the formation of ROS, which is induced by the accumulation of non-photoactive Pchlide, causes the necrotic phenotype in the *fgl* mutant leaves (Chakraborty and Tripathy 1992; op den Camp et al. 2003; Sakuraba et al. 2013). The biological functions of OsPORA and OsPORB are highly similar to those of HvPORA and HvPORB, respectively (Sakuraba et al. 2013). In addition, *OsPORB* is upregulated under high light treatment, suggesting that the function of *OsPORB* overlaps with that of *AtPORC* in Chl synthesis and maintenance (Sakuraba et al. 2013).

Although some species, including cucumber and pea, have only one POR, many photosynthetic plants have two or three isoforms of POR. Therefore, *por* mutants must be identified and characterized in every angiosperm to explore the biological diversity of POR members in detail, for example by studies of Arabidopsis POR isoforms (Frick et al. 2003; Masuda et al. 2003; Paddock et al. 2012; Sakuraba et al. 2013). In addition to studying *por* mutants, the functional redundancy of PORs has been examined by producing genetically modified Arabidopsis plants. Constitutive overexpression of *AtPORA* can restore the phenotypes of the *atporB atporC* double mutant (Paddock et al. 2010). In the current study, based on this finding, we explored whether *OsPORA* can fully substitute for *OsPORB* by producing transgenic rice plants constitutively overexpressing *OsPORA* in the *fgl* mutant background. We analyzed the transgenic plants under various growth conditions, finding that the function of *OsPORB* can be replaced by constitutively expressing *OsPORA* during vegetative growth under normal long days in paddy field conditions, but not under excessive light conditions or during reproductive growth. We discuss the physiological and developmental differentiation of the two rice PORs.

## Results

### Constitutive Overexpression of *OsPORA* in *fgl* Mutant Background Rescues Chl Synthesis and Leaf Development under Natural Field Conditions

**Fig. 1:**
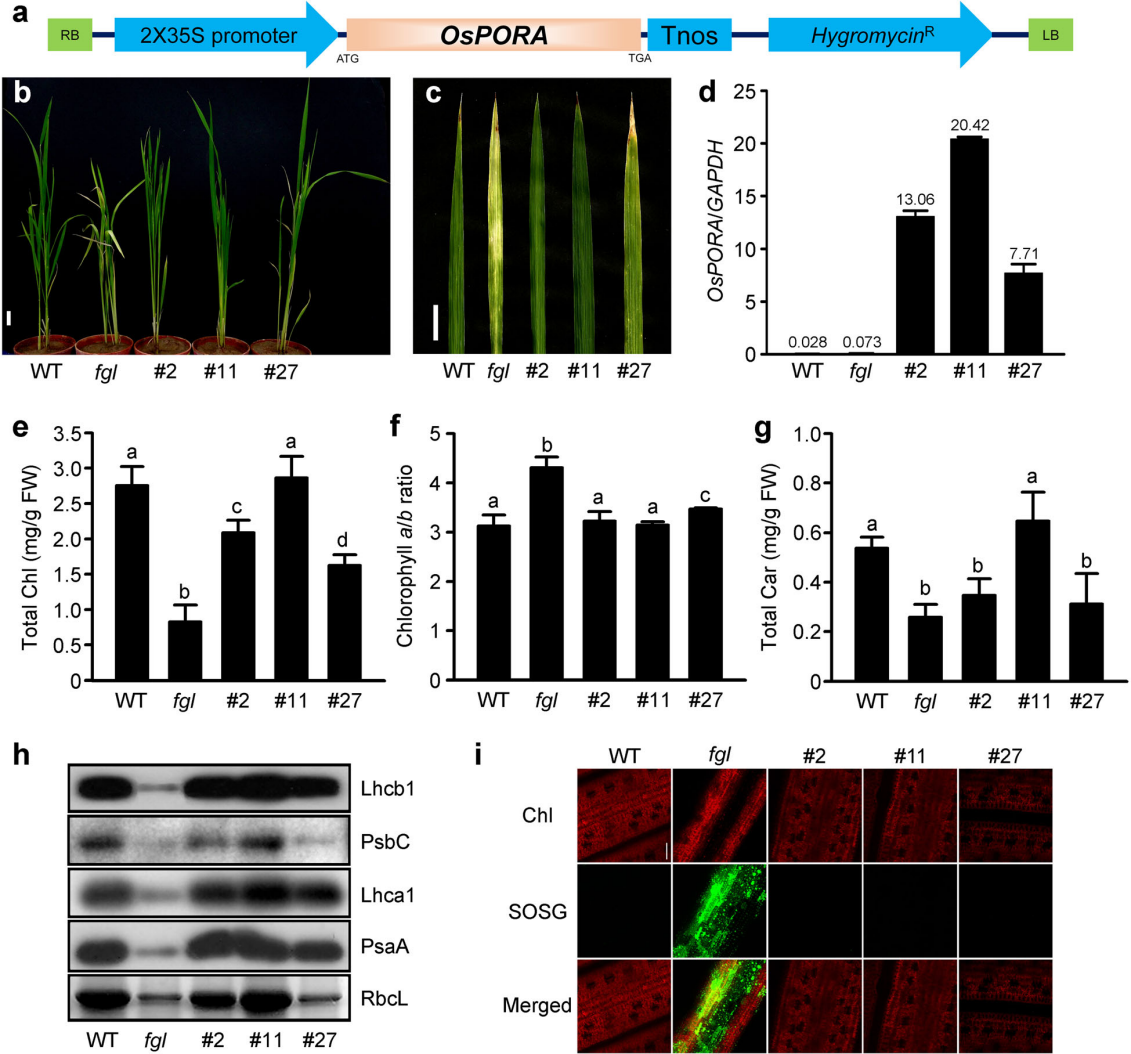
Characterization of *35S:OsPORA*/*fgl* (OPAO) plants grown under natural sunlight in the greenhouse. **a** Structure of the construct used to generate the OPAO transgenic plants. **b-c** Phenotypes of WT, *fgl*, and three independent OPAO T_0_ plants. Plants (**b**) and leaf tip regions of the fourth leaf blades of plants (**c**) grown in the greenhouse. Scale bars = 5 cm (**b**) and 2 cm (**c**). **d** Transcript levels of *OsPORA* in WT, *fgl*, and OPAO plant lines #2, #11, and #27. Means and standard deviations were obtained from three independent leaf parts. The numbers on the bars represent relative values from qRT-PCR. **e-g** Measurement of photosynthetic pigments in WT, *fgl*, and three OPAO lines. Total chlorophyll (Chl) contents (**e**), Chl *a*/*b* ratio (**f**), total carotenoid (Car) contents (**g**). The data were obtained from five independent leaf regions (**e-g**). **h** Immunoblot analysis of photosystem proteins. Lhcb1, PsbC, Lhca1, and PsaA were detected using specific antibodies, and Rubisco large subunit (RbcL) was detected by Coomassie Blue staining. Similar results were obtained in three independent experiments (**h**). **i** Detection of singlet oxygen (^1^O_2_) accumulation. Red indicates Chl autofluorescence (upper panels), green indicates SOSG fluorescence (middle panels), and merged Chl and SOSG fluorescent signals are shown (lower panels). Similar results were obtained from at least five independent leaf disks. Scale bar = 50 μm (**i**). Leaves were sampled from 2-month-old plants grown in the greenhouse. Letters a, b, and c on the bars indicate significantly different values at the 1% level according to Duncan’s multiple range test (**e-g**)

To examine the functional redundancy between *OsPORA* and *OsPORB*, we introduced *OsPORA* cDNA fused to the cauliflower mosaic virus *35S* promoter (*35S:OsPORA*) into *fgl* mutant by Agrobacterium-mediated transformation and generated 97 independent *35S:OsPORA/fgl* (hereafter, OPAO) transgenic T_0_ lines (Fig. 1a). Most OPAO plants exhibited normal leaf development under natural sunlight in the greenhouse compared with *fgl* mutant (data not shown). Among the OPAO lines, we selected three lines with different phenotypes based on leaf color: deep green (line #11), intermediate green (line #2), and light green (line #27) (Fig. 1b and c). Genomic PCR using specific primers, including regions of the *35S* promoter and *OsPORA*, amplified a single genomic DNA fragment from the transgenic lines (Additional file 1: Figure S1). We confirmed the expression levels of *OsPORA* mRNA in mature leaves of the transgenic lines. *OsPORA* is more highly expressed in the light green sectors of *fgl* leaves than in wild type (WT) (Sakuraba et al. 2013). We found the level of *OsPORA* mRNA in *fgl* mutant was approximately three times that of WT, and the mRNA levels of the three transgenic lines were over 100-fold higher than that of *fgl* mutant (Fig. 1d). In particular, the transcript level in line #11 was approximately 280-fold higher than that of *fgl* mutant (Fig. 1d). The following year, we generated T_1_ progeny from three transgenic T_0_ lines. The T_1_ plants were selected on the growth medium including hygromycin and transferred to a paddy field. The *OsPORA* mRNA levels of T_1_ plants (Additional file 2: Figure S2) were similar to those of T_0_ lines (Fig. 1d). The following year, homozygous T_2_ plants were selected by progeny testing on growth medium with hygromycin. The homozygous T_2_ progeny of line #11 were grown in the paddy field and showed a phenotypically rescued green leaf color, similar to WT (Additional file 3: Figure S3). These results suggest that overexpressing *OsPORA* can recover the defective leaf color phenotype of *fgl* mutant under natural growth conditions.

### *OsPORA* Overexpression Restores Photosynthetic Proteins and Pigments and Inhibits ROS Production in *fgl* Mutant

Next, we measured photosynthetic pigments in the leaf tissues of WT, *fgl*, and OPAO plants. Total Chl concentrations increased and the Chl *a*/*b* ratio decreased in the three OPAO lines compared to *fgl* mutant (Fig. 1e and f). The Chl level in OPAO line #11 was similar to that of WT, indicating that constitutive expression of *OsPORA* fully recovered Chl synthesis in *fgl* mutant background. Moreover, the total carotenoid content was fully recovered in line #11 (Fig. 1g). Similarly, the photosynthetic protein levels were higher in the OPAO lines than in *fgl* mutant (Fig. 1h). Thus, the levels of Chl synthesis and the formation of the photosynthetic apparatuses in the OPAO lines were nearly proportional to the expression levels of *OsPORA*. These results strongly suggest that OsPORA can substitute for OsPORB in Chl synthesis if *OsPORA* is constitutively expressed in developing leaves under natural growth conditions.

Yellow/white leaf variegation in *fgl* mutant is induced by excessive accumulation of ROS such as singlet oxygen (^1^O_2_) (Sakuraba et al. 2013). We thus examined the levels of singlet oxygen in the OPAO lines. In contrast to *fgl* mutant, singlet oxygen did not accumulate in the three OPAO lines (Fig. 1i), suggesting that the recovery of the necrotic lesion phenotype in these lines is related to the decrease in ROS.

### OPAO Plants Exhibit Normal Chloroplast and Etioplast Structure

**Fig. 2:**
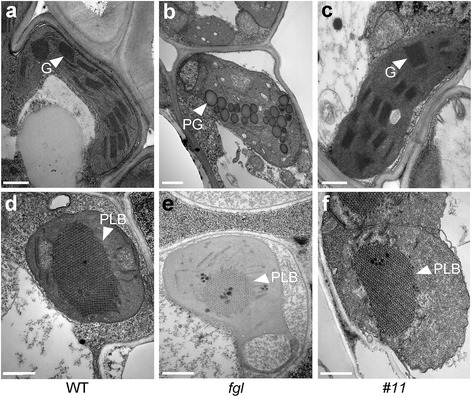
Ultrastructural analysis of plastids in the leaves of WT, *fgl*, and OPAO plants. **a-c** Chloroplasts in the WT leaf (**a**), the yellow sector of an *fgl* leaf (**b**), and the OPAO #11 homozygous T_2_ line leaf (**c**) grown for 80 days in the greenhouse. G, grana thylakoid; PG, plastoglobule. **d-f** Etioplasts in etiolated leaves of WT (**d**), *fgl* (**e**), and OPAO #11 line (**f**) grown in the dark for 10 days. PLB, prolamellar body. Scale bars = 0.5 μm (**a-f**). Similar results were obtained in at least three independent samples

In *fgl* mutant, the chloroplasts in both light green sectors and yellow/white sectors of *fgl* leaves display defective thylakoid stacking and have plastoglobules (Sakuraba et al. 2013). To investigate the chloroplast structure in OPAO plants, we sampled 80-day-old leaves from OPAO line #11 (Fig. 2a-c). The chloroplasts in the yellow/white sectors of *fgl* mutant were damaged and contained many plastoglobules and unstacked grana thylakoids (Fig. 2b). By contrast, the chloroplasts of WT and line #11 contained well-stacked grana thylakoids without plastoglobules (Fig. 2a and c), suggesting that the recovery of the leaf phenotype in OPAO plants is closely associated with the rescue of chloroplast development. Moreover, the restoration of thylakoid stacking by *OsPORA* overexpression might be related to the increased Chl concentration in *fgl* mutant (Fig. 1e). POR is involved in the production of prolamellar bodies (PLBs), which include carotenoids and Chl precursors in the etioplast (Rosinski and Rosen 1972; Engdahl et al. 2001), as the sizes of PLBs are reduced in the etioplasts of *por* mutants in both Arabidopsis and rice (Frick et al. 2003; Masuda et al. 2003; Paddock et al. 2012; Sakuraba et al. 2013). To examine whether PLBs are larger in the OPAO lines than in *fgl*, we observed the ultrastructures of etioplasts in 10-day-old T_2_ seedlings grown in dark conditions. The PLBs were much larger in OPAO line #11 than in *fgl* mutant but similar to those of WT (Fig. 2d-g). PLBs from line #11 also exhibited a well-organized lattice structure like that of WT (Fig. 2d and g). These results demonstrate that constitutive overexpression of *OsPORA* can fully recover the function of *OsPORB* during both etioplast and chloroplast development in developing and mature leaves in the absence of OsPORB activity.

### Overexpression of *OsPORA* Restores Normal Total and Photoactive Pchlide Levels

**Fig. 3:**
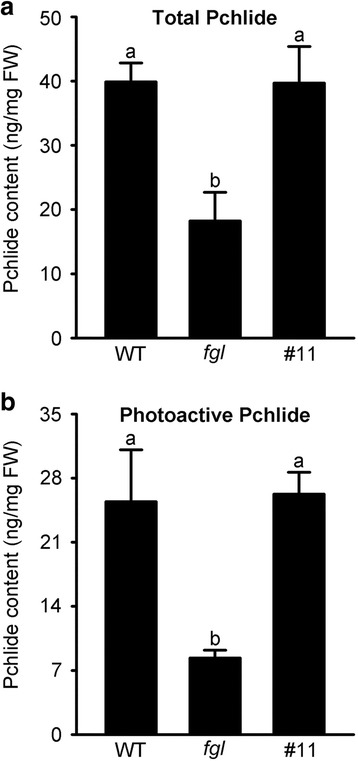
Levels of total Pchlide and photoactive Pchlide in WT, *fgl*, and the OPAO line. **a** Total Pchlide was measured based on fluorescence emission in pigments extracts from 10-day-old etiolated seedling grown in darkness. **b** Photoactive Pchlide levels were calculated by subtracting non-photoactive Pchlide from total Pchlide levels, which were measured in light-treated seedlings. The results were obtained and calculated from 10 independent etiolated leaves. The experiments were performed twice by fluorescence spectrophotometry, producing similar results. Letters a and b on the bars denote statistically significant differences based on Duncan’s test (*P* < 0.01)

Several studies in Arabidopsis and rice have suggested that PLB size is related to the levels of total and photoactive Pchlides (Franck et al. 2000; Paddock et al. 2010; Sakuraba et al. 2013). In particular, the levels of total and photoactive Pchlides in *fgl* mutant are significantly lower than those in WT, which is consistent with the reduced size of PLBs in *fgl* mutants (Sakuraba et al. 2013). We therefore collected 10-day-old etiolated WT, *fgl*, and OPAO line #11 seedlings and measured their total and photoactive Pchilde levels using fluorescence and UV/VIS spectrophotometry (Fig. 3 and Additional file 4: Figure S4). As previously shown, both total and photoactive Pchlide levels were markedly reduced in *fgl* mutant compared to WT (Fig. 3). However, the concentrations of total and photoactive Pchlides in OPAO line #11 increased to the WT levels (Fig. 3). Furthermore, the ratio of photoactive to total Pchlides was approximately 0.63 in WT, 0.45 in *fgl*, and 0.66 in line #11, suggesting that the photoactive Pchlide level was completely recovered in etiolated seedlings of the OPAO line. We obtained similar results from UV/VIS spectrophotometry of Pchlide levels in WT, *fgl*, and line #11 plants (Additional file 4: Figure S4). These data suggest that in the absence of OsPORB, *OsPORA* overexpression leads to the formation of photoactive Pchlide for normal Chl synthesis during very early rice development.

### OPAO Plants Show Partially Rescued Chl and Carotenoid Contents under Constant Light Conditions

**Fig. 4:**
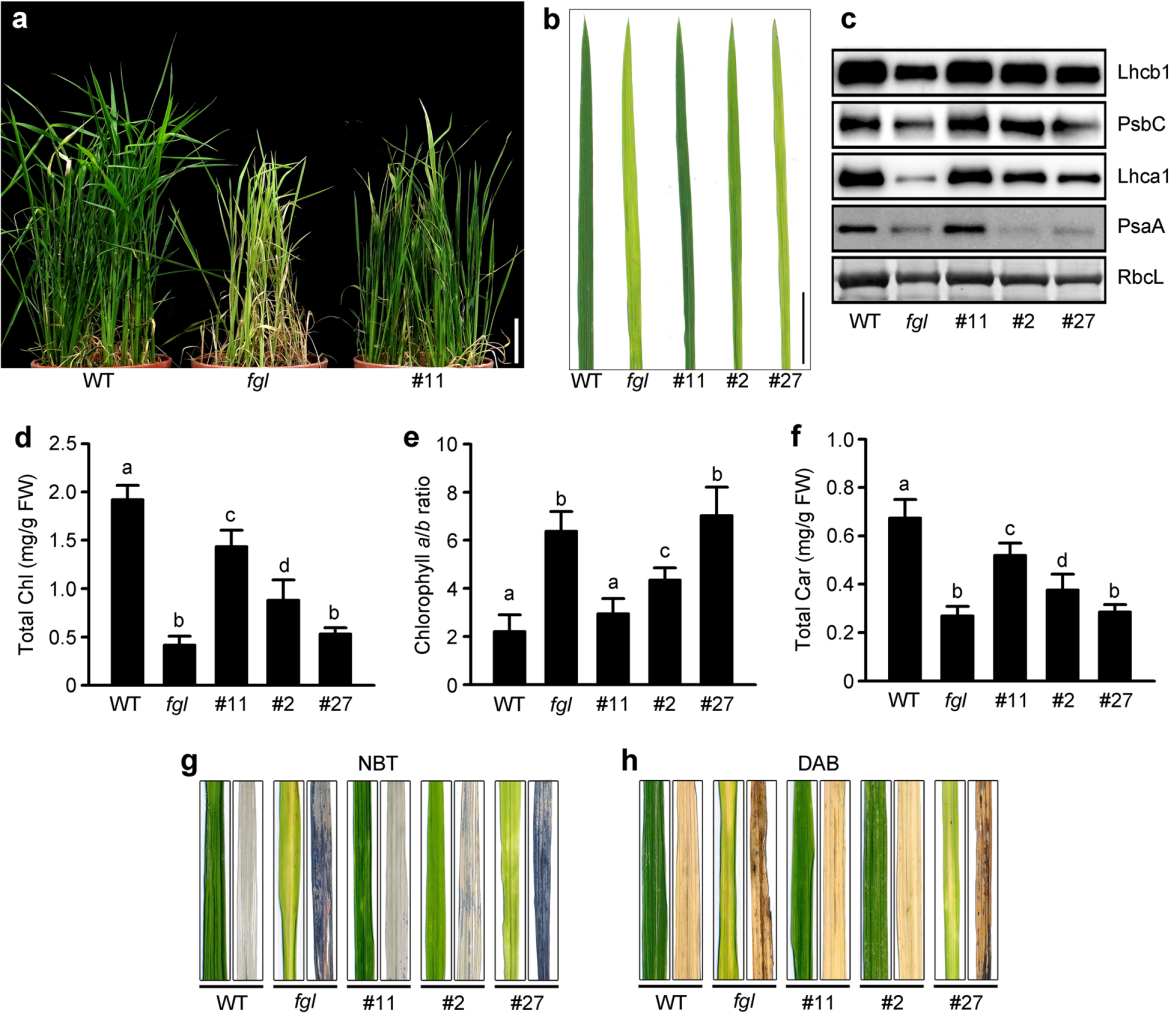
Characterization of OPAO plants under CL conditions. **a-b** Phenotypes of WT, *fgl*, and OPAO homozygous T_2_ plants. Shoots (**a**) and third leaf blades (**b**) grown under CL conditions in an artificial growth chamber (200 μmol m^−2^ s^−1^). Scale bar = 2 cm (**b**). **c** Immunoblot analysis of photosystem proteins. The proteins were detected using specific antibodies, but RbcL was visualized by Coomassie Blue staining (**c**). The detection was repeated three times with independent biological replicates (**c**). **d-f** Quantification of photosynthetic pigments in WT, *fgl*, and OPAO line #11. Total Chl contents (**d**), Chl *a*/*b* ratio (**e**), total Car contents (**f**). The data were obtained from 10 independent leaf samples, and means with different letter are significantly different (Duncan’s test, *P* < 0.01) (**d-f**). **g-h** Determination of ROS accumulation in leaves. Superoxide anion radicals (O_2_^−^) (**g**) and hydrogen peroxide (H_2_O_2_) (**h**) in leaves. Superoxide anion radicals and hydrogen peroxide were visualized by staining with NBT (**g**) and DAB (**h**), respectively. Samples were obtained from 2-week-old plants grown in a growth chamber. All analyses were repeated at least twice and showed similar results

*OsPORA* is dark-induced and expressed at early stages of leaf development; also, OsPORA contributes to light-dependent Chl synthesis and inhibits the formation of necrotic lesions during early leaf development in the presence of OsPORB (Sakuraba et al. 2013). Under constant light (CL) conditions, *fgl* mutant exhibited severe leaf variegation and repressed Chl synthesis (Sakuraba et al. 2013); the rapid decrease in *OsPORA* expression may be the main reason for the defective phenotype. Thus, to investigate whether constitutive overexpression of *OsPORA* could compensate for the effects of *OsPORB* deficiency on Chl synthesis under CL conditions, we grew WT, *fgl*, and line #11 plants for 2 weeks under CL conditions and determined their leaf phenotypes and levels of photosynthetic pigments. Two-week-old OPAO line #11 plants had normal green leaves with no necrotic lesions, which is similar to the WT phenotype, indicating that ectopic expression of *OsPORA* rescues leaf development in the absence of OsPORB, even under CL conditions (Fig. 4a).

Next, we analyzed the phenotypes of the other OPAO lines (line #2 and #27; Fig. 1) in more detail. The three OPAO lines had different levels of *OsPORA* mRNA (Fig. 1d and Additional file 2: Figure S2) and displayed dark (line #11), intermediate (line #2), and light green (line #27) leaf color (Fig. 4b). In line #27, which had the lowest *OsPORA* mRNA level among OPAO lines, the leaf color and photosynthetic protein levels were similar to those of *fgl* mutant (Fig. 4b and c). We also examined total Chl contents, Chl *a*/*b* ratios, and total carotenoid levels in leaves (Fig. 4d-f). In contrast to the results under natural conditions, the total Chl and carotenoid contents in the three OPAO lines did not recover to WT levels (Fig. 4d and f). In particular, the photosynthetic pigment levels in line #27 were similar to those in *fgl* mutant, indicating that *OsPORA* expression in line #27 is below the threshold level required to recover Chl synthesis in the *fgl* mutant background (Fig. 4d-f). Interestingly, in line #11, with the highest *OsPORA* mRNA level, the Chl and carotenoid levels did not fully recover to WT levels (Fig. 4d and f). We then performed phenotypic characterization of line #11 plants grown under short day (SD) conditions compared to CL conditions (Additional file 5: Figure S5). Under SD conditions, all leaf-associated phenotypes of line #11 completely recovered to those of WT, in contrast to the results under CL conditions (Additional file 5: Figure S5). This result strongly suggests that Chl synthesis in the absence of OsPORB cannot be completely restored during early development under CL conditions, even though *OsPORA* mRNA levels are extraordinarily high.

Abnormally high levels of ROS cause the lesion formation and leaf variegation phenotypes in *fgl* mutant (Sakuraba et al. 2013). Some leaves in line #27 showed variegation and contained ROS such as hydrogen peroxide (H_2_O_2_) and superoxide anion radicals (O_2_^·-^), as was observed in the leaf tissues of *fgl*, but this was not the case in lines #11 and #2 (Fig. 4g and h). The leaf variegation of line #27 under CL conditions is likely caused by ROS accumulation to levels corresponding to those found in *fgl* mutants.

### *OsPORA* Expression in OPAO Plants is Consistent under CL and SD Conditions

**Fig. 5:**
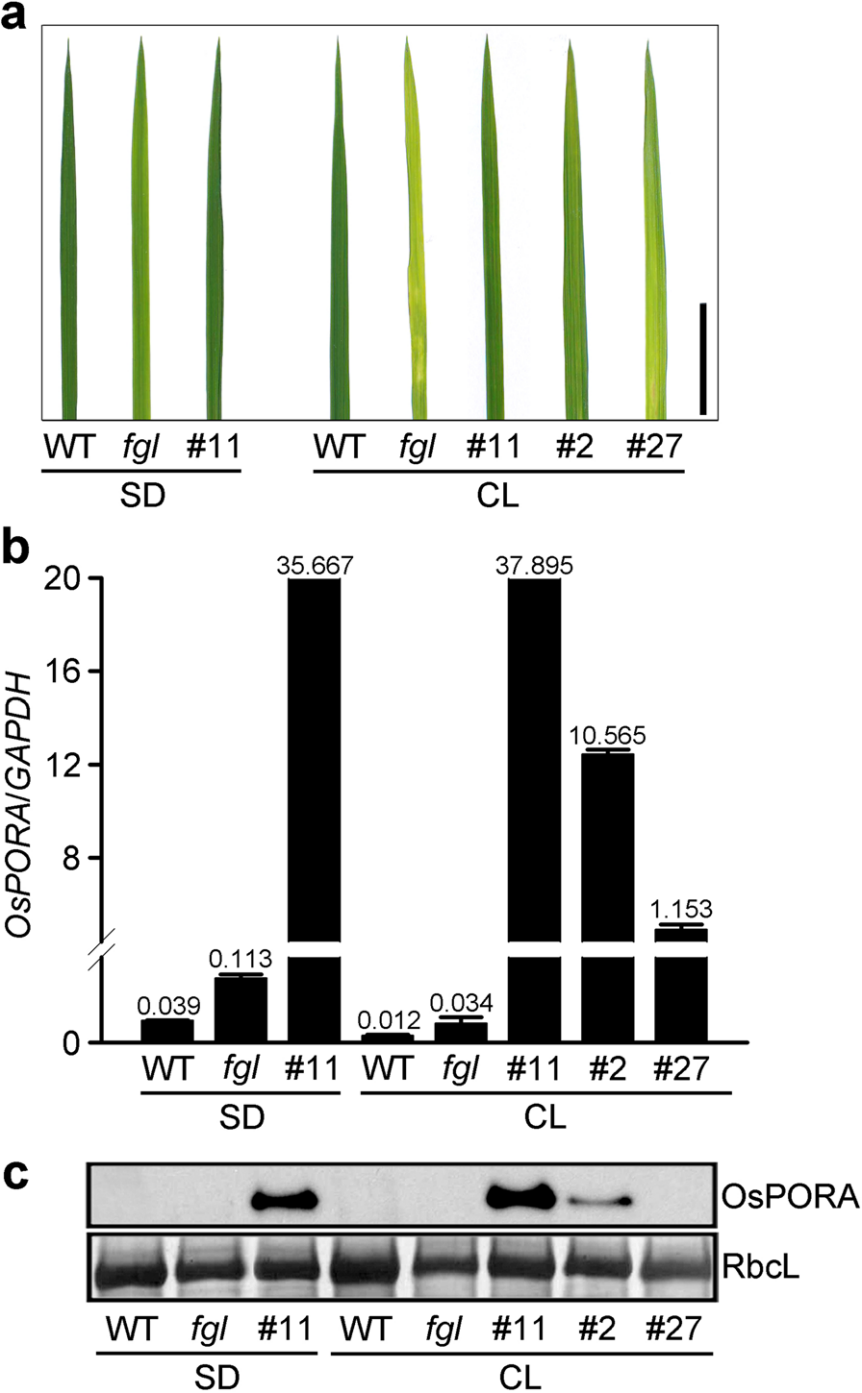
Expression patterns of *OsPORA* transcript and OsPORA protein in WT, *fgl*, and OPAO lines under SD and CL conditions. **a** Phenotypes of third leaf blades. Plants were grown for 2 weeks in artificial growth chambers (200 μmol m^−2^ s^−1^). Scale bar = 2 cm (**a**). **b** Transcript levels of *OsPORA* in WT, *fgl*, and OPAO plants. Numbers on the bars indicate relative values quantified by RT-qPCR. Means and standard deviations were obtained from three independent leaf blades (**b**). **c** Immunoblot assay of OsPORA protein. RbcL was visualized using CCB staining as a control (**c**). All analyses were repeated at least twice and produced similar results

The expression of *OsPORA* decreases markedly under CL and high-light conditions. In addition, OsPORA protein levels are proportional to its transcript levels under various light conditions, indicating that OsPORA protein levels are regulated at the transcriptional level (Sakuraba et al. 2013). We therefore investigated whether the compromised rescue of the OPAO lines is related to changes in *OsPORA* mRNA or OsPORA protein levels. We grew the OPAO plants under SD and CL conditions and analyzed their *OsPORA* mRNA and OsPORA protein levels (Fig. 5). In contrast to our hypothesis, OPAO line #11 showed almost the same levels of *OsPORA* mRNA and OsPORA protein under both SD and CL conditions (Fig. 5b and c). Furthermore, the levels of OsPORA protein in OPAO line #11 were consistent with its transcriptional pattern (Fig. 5b and c). These results indicate that the *OsPORA* mRNA and OsPORA protein levels in this line are not dependent on photoperiod.

### Recovery of the Expression of Photosynthesis- and Chl Synthesis-Related Genes in the OPAO Lines

**Fig. 6:**
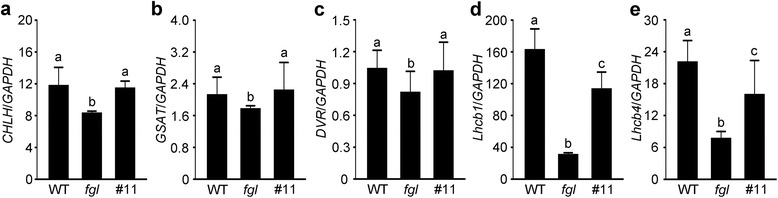
Transcriptional analysis of Chl synthesis- and photosynthesis-related genes in WT, *fgl*, and OPAO plants. **a-e** Relative transcript levels of *CHLH* (**a**), *GSAT* (**b**), *DVR* (**c**), *Lhcb1* (**d**), and *Lhcb4* (**e**) in leaf blades of plants grown under CL conditions for 2 weeks. *CHLH* encodes Mg-chelatase H subunit (Os03g20700); *GSAT*, glutamate-1-semialdehyde aminotransferase (Os08g41990); *DVR*, 3,8–divinyl chlorophyllide *a* 8–vinyl reductase (Os03g22780); *Lhcb1*, light-harvesting Chl *a*/*b*-binding protein of photosystem II (Os01g41740); *Lhcb4* (Os07g37240). Averages and standard deviations were determined using three biological replicates. Lower-case letters on the bars denote significantly different values determined by Duncan’s multiple range test (*P* < 0.01). The experiments were conducted at least twice, showing similar results

Several photosynthesis- and Chl synthesis-associated genes were previously found to be downregulated in the green sectors of 2-week-old *fgl* leaves under CL conditions (Sakuraba et al. 2013). To investigate the molecular basis for the recovery of the OPAO lines, we examined the transcriptional patterns of two photosynthesis-related genes (*Lhcb1* and *Lhcb4*) and three genes involved in Chl synthesis (*CHLH*, *GSAT*, and *DVR*) in the leaves of plants grown under CL conditions (Fig. 6). The expression levels of the five genes were higher in OPAO line #11 than in *fgl* mutant, indicating that leaf recovery in the OPAO lines is closely associated with an increase in the expression levels of these genes (Fig. 6). However, the expression of *Lhcb1* and *Lhcb4* was not restored to the WT levels (Fig. 6c-e). Perhaps the partial upregulation of these genes is associated with the incomplete rescue of the OPAO lines under CL conditions.

### Compromised Rescue of OPAO Plants under Natural Field Conditions Beginning at the Early Reproductive Phase

**Fig. 7:**
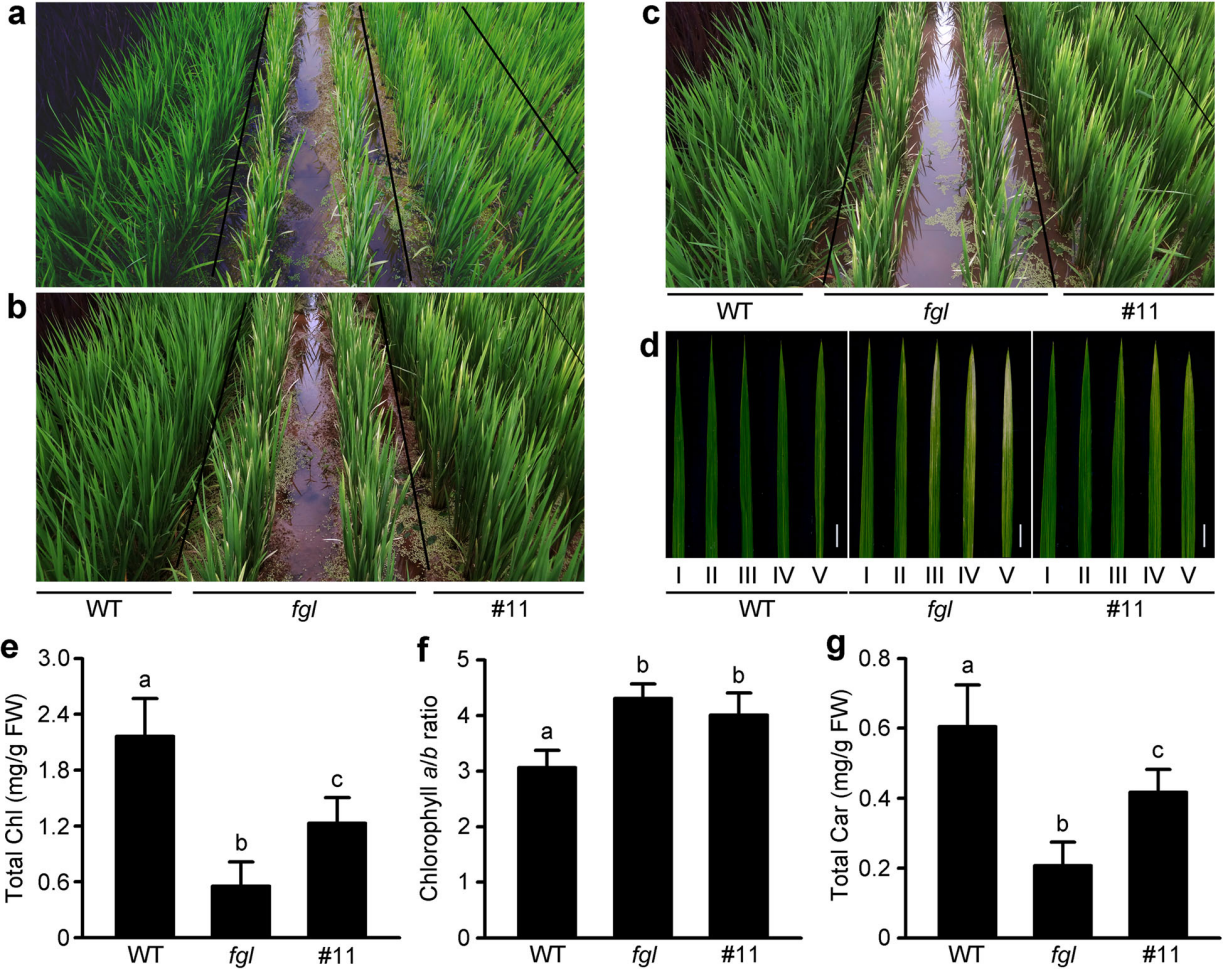
Phenotypic characterization of WT, *fgl*, and OPAO T_2_ plants grown under natural paddy field conditions. **a-c** Plants in a paddy field at 90 (**a**), 95 (**b**), and 100 (**c**) days after sowing (DAS). **d** Leaf blades of the main culms. Samples were obtained from the fourth leaves of plants at 105 DAS. Roman numerals below the panels indicate leaf position: e.g. I, youngest leaves (upper position); V, oldest leaves (lower position). Scale bar = 2 cm (**d**). **e-g** Total Chl contents (**e**), Chl *a*/*b* ratio (**f**), and total Car contents (**g**). The pigments were extracted from distal region of the fourth leaves at 105 DAS. The data were obtained from 10 independent samples, and different letters above the bars indicate significantly different values (Duncan’s test, *P* < 0.01) (**e-g**)

Overexpressing *AtPORA* in the Arabidopsis *atporB atpocC* double mutant fully restores Chl synthesis during plant growth (Paddock et al. 2010). However, we detected degreening of leaf tips in OPAO plants beginning at the early reproductive stage. The distal regions of leaf blades in OPAO line #11 plants grown in a natural paddy field turned light green beginning at 90 days after sowing (Fig. 7a). This phenomenon became severe until heading and flowering (Fig. 7a-d). The degreening process began in older leaves of *fgl* mutant, and OPAO leaves showed an intermediate phenotype between WT and *fgl* (Fig. 7d). We analyzed photosynthetic pigment levels in the leaves of WT, *fgl*, and line #11 plants (Fig. 7e-g). Total Chl and carotenoid contents were higher in the OPAO line than in *fgl* mutant and lower than in WT (Fig. 7e and g), which is consistent with their leaf phenotypes (Fig. 7d). In addition, the Chl *a*/*b* ratio in line #11 was higher than that of WT, as was that in *fgl* mutant (Fig. 7f). These results suggest that OsPORA activity can compensate for the absence of OsPORB activity during the juvenile and vegetative stages of plant growth. However, after the vegetative phase, the role of OsPORA in Chl synthesis is limited, even if *OsPORA* is constitutively overexpressed.

## Discussion

In the model dicot plant Arabidopsis, three homologous *POR* genes encode structurally similar enzymes that are synthesized as pro-proteins and modified in the plastid (Franck et al. 2000; Frick et al. 2003; Masuda et al. 2003; Paddock et al. 2010; Reinbothe et al. 2015). Even though the sequences of PORs are highly conserved, the mature POR isoforms play somewhat distinct roles during development due to their dissimilar expression patterns (Franck et al. 2000; Frick et al. 2003; Masuda et al. 2003; Paddock et al. 2010; Paddock et al. 2012). Rice contains two POR proteins, OsPORA and OsPORB, which have distinct functions during leaf growth. For example, OsPORA functions in the early stage of leaf development, while OsPORB functions throughout development (Sakuraba et al. 2013). In the current study, by producing transgenic rice plants overexpressing *OsPORA* in the *fgl* mutant background, we found that the functional deficiency of *OsPORB* could be overcome by the ectopic expression of *OsPORA*. Under CL conditions, however, the Chl contents of the OPAO lines during early development were lower than those of WT (Fig. 4d), in contrast to the results obtained under SD conditions in growth chambers (Additional file 5: Figure S5e) or natural long day conditions in the greenhouse (Fig. 1e). Because the expression levels of *OsPORA* mRNA and protein in OPAO line #11 were highly upregulated under both SD and CL conditions (Fig. 5b and c), the partial rescue of the mutant phenotype under CL conditions might be related to the lack of OsPORB, indicating that functional variation exists between *OsPORA* and *OsPORB*.

The Arabidopsis *porA* mutants show severe deficiencies in photoautotrophic development, as well as diminished photoactive Pchlide conversion and reduced prolamellar body volume during skotomorphogenesis (Paddock et al. 2012). In addition, the elimination of AtPORA activity during photomorphogenesis results in reduced total Chl contents and abnormal plant growth, although *AtPORA* is transiently expressed during illumination (Armstrong et al. 1995; Paddock et al. 2012). However, the function of *OsPORA* in rice is still unclear, since no reports about *osporA*-deficient mutants are currently available. The rice *fgl* mutant shows milder defects than Arabidopsis *atporB atporC* double mutants (Frick et al. 2003; Sakuraba et al. 2013). Because *OsPORA* is highly expressed in developing leaves at the early seedling stage, the function of OsPORA is considered to be essential for Chl synthesis in rice leaves (Sakuraba et al. 2013). Our observation of OPAO lines suggests that *OsPORA* functions in Chl synthesis in leaves, even after early development (Fig. 1 and Additional file 3: Figure S3). Our findings imply that the role of *OsPORA* in rice development is generally controlled by its expression pattern. However, OPAO T_2_ plants grown under natural field conditions exhibited partially recovered phenotypes in leaf blades beginning at the booting stage (before the heading stage), i.e., approximately 90 days after sowing (Fig. 7a). Finally, the leaf phenotype of the OPAO lines was intermediate between WT and *fgl* mutant (Fig. 7d-g), suggesting that beginning at the reproductive phase, Chl synthesis is not completely controlled only by OsPORA, although *OsPORA* expression levels are quite high. Previous and current findings suggest that the difference between the activities of OsPORA and OsPORB might at least be partially attributed to the different spatial and temporal expression patterns of the two *OsPORs*, as well as their differing protein functions.

A reduction in PLB size leads to reduced total Pchlide levels (Franck et al. 2000; Sakuraba et al. 2013). The PLB size in the OPAO lines was recovered to that of WT, leading to an increase in total Pchlide contents (Fig. 2d-g and 3a). In addition to increased total Pchlide levels, photoactive Pchlide in the OPAO lines accumulated to WT levels (Fig. 3b). This result indicates that although maintaining threshold levels of Pchlide formation in etiolated seedlings appears to require OsPORB activity, ectopic expression of *OsPORA* is sufficient for rescuing total and photoactive Pchlide levels in *fgl* mutant. In other words, normal levels of OsPORA in the absence of OsPORB activity are not sufficient for the formation of enough Pchlide to maintain persistent green coloration in mature leaves. Thus, the enzymatic functions of the two OsPORs are redundant during the juvenile growth stage. Moreover, POR-mediated conversion from Pchlide to Chlide is completely light dependent (Griffiths 1978). Pchlide bound to POR is referred to as “photoactive”, whereas Pchlide that is not bound by POR, which is referred to as “non-photoactive”, readily functions as a photosensitizer to produce singlet oxygen, thereby inducing photo-oxidative damage (Matringe et al. 1989; Reinbothe et al. 1996; Sperling et al. 1997; Mock et al. 1998; Heyes and Hunter 2005). In addition, HvPORA plays a photo-protective role during greening through the reconstitution of the light-harvesting POR: Pchlide complex (Buhr et al. 2008). The leaf variegation in *fgl* mutant might be caused by photosensitization induced by the accumulation of high levels of non-photoactive Pchlide (Sakuraba et al. 2013). Therefore, the rescue of necrotic lesions in the OPAO lines is closely related to the disappearance of ROS, including singlet oxygen, generated from non-photoactive Pchlide (Figs. 1i and 4g-h). In addition, ROS accumulation in *fgl* chloroplasts likely provokes the downregulation of Chl synthesis and photosynthetic genes in the nucleus via retrograde signaling from the chloroplast (Gadjev et al. 2006; Sakuraba et al. 2013). Indeed, the transcript levels of photosynthesis-associated genes (*Lhcb1* and *Lhcb4*), as well as upstream (*GSAT* and *CHLH*) and downstream (*DVR*) genes of *POR*, were rescued in OPAO line #11 (Fig. 6). These results suggest that the recovery of necrotic lesions in the OPAO lines is caused by the disappearance of ROS derived from the reduction in non-photoactive Pchlide levels due to *OsPORA* overexpression, which upregulates these genes, thereby leading to the removal of oxidative damage.

OsPORA and OsPORB protein levels are directly proportional to their transcript levels, suggesting that OsPOR activity is mainly controlled at the transcriptional level (Sakuraba et al. 2013). In particular, the levels of *OsPORA* mRNA and protein are rapidly downregulated under long periods and high levels of illumination (Sakuraba et al. 2013). Similarly, Arabidopsis *PORA* (*AtPORA*) mRNA is barely detectable in leaf tissues of plants grown in the light, in contrast to *AtPORB* and *AtPORC* mRNA levels (Armstrong et al. 1995; Su et al. 2001). In the OPAO lines, we predicted that *OsPORA* would be constitutively expressed throughout development. As expected, *OsPORA* transcript levels in OPAO line #11 were consistent under both SD and CL conditions (Fig. 5b). Moreover, OsPORA protein levels did not differ between SD and CL conditions (Fig. 5c), indicating that excessive light does not affect the protein stability of OsPORA. However, Chl synthesis in the OPAO plants was not completely restored under CL conditions (Fig. 6d), although the levels of OsPORA protein remained high independent of light period. Hence, the compromised recovery of leaf greenness in the OPAO lines under CL conditions may be due to the weakening of OsPORA activity upon exposure to excessive light, and thus, the overall POR activity drops below the threshold level, which is not sufficient for producing Pchlide for Chl synthesis in the absence of light-stable OsPORB activity.

Many studies have investigated the possibility of the functional redundancy of the POR isoforms, as their exact functions in plant development are currently unclear, although their sequences are highly conserved (Sperling et al. 1998; Franck et al. 2000; Masuda et al. 2003; Frick et al. 2003; Paddock et al. 2010; Paddock et al. 2012). The amino acid sequences of OsPORA and OsPORB, the two POR isoforms in rice, are remarkably similar, and POR enzymes are highly conserved among angiosperms (Sakuraba et al. 2013). Because of this sequence conservation, we hypothesized that the two rice POR proteins are functionally equivalent, despite their distinct expression patterns. The Arabidopsis *atporB atpocC* double mutants are totally recovered by ectopic expression of *AtPORA* (Paddock et al. 2010). In the current study, however, overexpressing *OsPORA* in plants lacking *OsPORB* gave incomplete rescue of leaves, but only under CL conditions at the early vegetative stage and beginning at the early reproductive phase under natural field conditions (Figs. 4 and 7). Both lesion formation and degreening in the *fgl* mutant under natural field conditions (beginning at the tips of older leaves) are caused by the suppression of *OsPORA* expression*,* resulting in the complete absence of POR activity (Sakuraba et al. 2013). In this study, however, we found that in developing leaves under excessive light conditions and in mature leaves beginning at the reproductive stage, even high levels of *OsPORA* mRNA and protein failed to fully substitute for OsPORB activity, which is required for normal levels of Chl synthesis.

## Conclusions

OsPORs, which are essential for Chl synthesis, are present in two isoforms in rice, but the biological diversity of their roles has been unclear. We generated transgenic plants to investigate the functional differences between OsPORA and OsPORB under various growth conditions. Although the constant presence of OsPORA activity restored the leaf phenotype of *fgl* mutant, the function of OsPORB was not fully replaced by OsPORA activity under certain growth conditions. Taken together, our findings indicate that the functional differentiation of the two rice PORs is closely associated with maintaining the photosynthetic activity of leaves until senescence. Thus, we propose that the two OsPORs have differentiated during evolution to play distinct roles in the adaptation of rice to the environment.

## Methods

### Plant Materials and Growth Conditions

The single recessive *fgl* mutant was previously generated from *japonica* rice cultivar ‘Kinmaze’ by methyl nitrosourea (MNU) mutagenesis as previously described (Iwata and Omura 1975). For phenotypic characterization, plants were grown under natural long days (~14 h light/day) in a paddy field (Suwon, Republic of Korea, 37° N latitude), greenhouse (Seoul, Republic of Korea), and growth chambers. The chamber conditions were: 24 h light at 30 °C for CL and 10 h light at 30 °C/14 h dark at 24 °C for SD with 70% relative humidity. Light-emitting diodes (LEDs) were used and the average photon flux density was 200 μmol m^−2^ s^−1^.

### Vector Construction and Rice Transformation

*OsPORA* cDNA was obtained by reverse-transcription polymerase chain reaction (RT-PCR) with total RNA extracted from the leaves of *japonica* rice cultivar ‘Nipponbare’ using gene-specific primers (Additional file 6: Table S1). *OsPORA* was ligated into the pCR8/GW/TOPO plasmid (Invitrogen). To overexpress *OsPORA* in *fgl* mutant, full-length *OsPORA* cDNA was subcloned into the pMDC32 Gateway binary vector containing the cauliflower mosaic virus *35S* promoter (Curtis and Grossniklaus 2003). The recombinant plasmid was introduced into calli generated from mature embryos from *fgl* mutant seeds through *Agrobacterium* (strain EHA105)-mediated transformation (Jeon et al. 2000). Transgenic plants were grown in Murashige and Skoog medium for 2 weeks and transferred to soil. Vector insertion in the transgenic plants was confirmed by PCR with primers for the pMDC32 vector and *OsPORA* fragment (Additional file 6: Table S1).

### Measurement of Photosynthetic Pigments

To evaluate total chlorophyll and carotenoid concentrations, pigments were extracted from leaves with 80% acetone. Chlorophyll and carotenoid levels were measured with a UV/VIS spectrophotometer (BioTek) as described previously (Lichtenthaler 1987). Total Pchlide was extracted from leaf blades with 80% acetone containing 0.1 N ammonium hydroxide at 4 °C. Total Pchlide was measured in etiolated seedlings grown in the dark for 10 days. Photoactive Pchlide levels were calculated based on the difference between total Pchlide and non-photoactive Pchlide, which was determined in illuminated etiolated seedlings. Pchlide and photoactive Pchlide concentrations were calculated using a Cary Eclipse fluorescence spectrophotometer (Varian) or UV/VIS spectrophotometer (BioTek). The slit widths of both excitation and emission in the fluorescence spectrophotometer were set at 10 nm, and the excitation and emission wavelengths were 433 nm and 634 nm, respectively. The values from fluorescence spectrophotometry were calibrated using an absorption coefficient of 30.4 mM^−1^ cm^−1^ (Brouers and Michel-Wolwertz 1983; Sperling et al. 1998). UV/VIS spectrophotometry was performed as described previously (Brouers and Michel-Wolwertz, 1983). All experiments were performed as previously described (Anderson and Boardman 1963; Brouers and Michel-Wolwertz 1983; Sperling et al. 1998; Masuda et al. 2003; Paddock et al. 2010; Paddock et al. 2012).

### Transmission Electron Microscopy (TEM) Analysis

Leaf samples for TEM analysis were harvested from 3-month-old plants grown in the greenhouse and from 10-day-old dark-grown etiolated seedlings. Whole tissue preparation was carried out as described previously (Park et al. 2007). Segments of leaf tissues were fixed in modified Karnovsky’s fixative (2% paraformaldehyde, 2% glutaraldehyde, and 50 mM sodium cacodylate buffer, pH 7.2) and washed three times with 50 mM sodium cacodylate buffer, pH 7.2, at 4 °C for 10 min. The samples were post fixed with 1% osmium tetroxide in 50 mM sodium cacodylate buffer, pH 7.2, at 4 °C for 2 h and briefly washed twice with distilled water at 25 °C. The samples were en bloc stained in 0.5% uranyl acetate at 4 °C for a minimum of 30 min, dehydrated in a gradient series of ethanol and propylene oxide, and embedded in Spurr’s resin. After polymerization at 70 °C for 24 h, the sections were sliced to 60 nm with an ultramicrotome (MT–X; RMC) and stained with 2% uranyl acetate for 5 min and Reynolds’ lead citrate for 2 min at 25 °C. The processed samples were then examined under a JEM-1010 EX electron microscope (JEOL).

### ROS Analysis

Detection of singlet oxygen was conducted as previously described with some modifications (Sakuraba et al. 2013). For singlet oxygen staining, 3-month-old leaves were infiltrated in the dark with a solution of 200 μM Singlet Oxygen Sensor Green reagent (SOSG, Invitrogen) in 50 mM sodium potassium buffer (pH 7.5). After 30 min incubation, the leaf discs were washed once with 50 mM sodium potassium buffer and three times in distilled water. The fluorescence emission of SOSG was detected by confocal laser scanning microscopy (LSM510, Carl Zeiss). The excitation and emission wavelengths were 480 nm and 520 nm, respectively. Detection of hydrogen peroxide and superoxide by 3,3-diaminobendizine (DAB) and nitroblue tetrazolium chloride (NBT) staining, respectively, was performed as previously described (Han et al. 2012).

### Sodium Dodecyl Sulfate Polyacrylamide Gel Electrophoresis (SDS-PAGE) and Immunoblot Analysis

Photosynthesis-related and OsPOR proteins were detected as previously described, with some modifications (Han et al. 2012; Kwon et al. 2015a). Leaf tissue (10 mg) was homogenized in 100 μl of SDS sample buffer (50 mM Tris, pH 6.8, 2 mM EDTA, 10% w/v glycerol, 2% SDS, and 6% β-mercaptoethanol), and the extracted proteins were denatured at 100 °C for 3 min, resolved by SDS-PAGE, and immunoblotted. All antibodies were obtained from Agrisera. Rubisco large subunit (RbcL) was visualized by staining with Coomassie Brilliant Blue reagent (Sigma-Aldrich).

### RT-PCR and Quantitative Reverse-transcription PCR (qRT-RCR)

RT-PCR and qRT-PCR were carried out as previously described with slight modifications (Kwon et al. 2015b). Total RNA was extracted from leaves using a Total RNA Extraction Kit (MGmed, Seoul, Republic of Korea). First-strand cDNA was synthesized from 2 μg total RNA using oligo(dT)_15_ primer and M-MLV reverse transcriptase (Promega). The transcript levels of genes were detected using gene-specific primers (Additional file 6: Table S1). *Glyceraldehyde phosphate dehydrogenase* (*GAPDH*) was used as an internal control (Additional file 6: Table S1). The 20 μl reactions included 2 μl of 0.5 μM primer, 2 μl of cDNA mixture, and 10 μl of 2X GoTaq qPCR Master Mix (Promega). PCR was performed with a Light Cycler 2.0 instrument (Roche) using the following program: 94 °C for 2 min, 40 cycles of 94 °C for 15 s and 60 °C for 1 min.

## Additional files

Additional file 1: Figure S1. PCR-based confirmation of *OsPORA* insertion in genomic DNA of three independent OPAO T0 lines. (PDF 136 kb)
Additional file 2: Figure S2. Expression levels of *OsPORA* in the 2nd leaves of OPAO T1 lines. (PDF 125 kb)
Additional file 3: Figure S3. Phenotypes of WT, *fgl* mutant, and OPAO homozygous T2 line #11 in the paddy field. (PDF 573 kb)
Additional file 4: Figure S4. Total Pchlide and photoactive Pchlide levels determined by UV/VIS spectrophotometry. (PDF 175 kb)
Additional file 5: Figure S5. Phenotypic characterization of OPAO line #11 under SD conditions. (PDF 339 kb)
Additional file 6: Table S1. Primers used in this study. (PDF 125 kb)

## Abbreviations

Chl: Chlorophyll
ROS: Reactive oxygen species
Pchlide: Protochlorophyllide
Chlide: Chlorophyllide
POR: NADPH: protochlorophyllide oxidoreductase
PLB: Prolamellar body
LPOR: Light-dependent POR
DPOR: Dark-operative POR
*fgl*: *faded green leaf*
WT: Wild type
OPAO: *OsPORA*-overexpressing
CL: Constant light
SD: Short days
MNU: Methyl nitrosourea
LEDs: Light-emitting diodes
TEM: Transmission electron microscopy
SOSG: Singlet oxygen sensor green
DAB: 3,3-Diaminobendizine
NBT: Nitroblue tetrazolium chloride
SDS-PAGE: Sodium dodecyl sulfate polyacrylamide gel electrophoresis
RbcL: Rubisco large subunit
GAPDH: Glyceraldehyde phosphate dehydrogenase
Car: Carotenoid
CHLH: Mg-chelatase H subunit
GSAT: Glutamate-1-semialdehyde aminotransferase
DVR: 3,8–divinyl chlorophyllide *a* 8–vinyl reductase
Lhcb: light-harvesting Chl *a*/*b*-binding protein of photosystem II
Lhca: light-harvesting Chl *a*/*b*-binding protein of photosystem I
PsbC: Photosystem II protein C
PsaA: Photosystem I protein A
DAS: Days after sowing
