# Supplementary material for: Two NADPH: Protochlorophyllide Oxidoreductase (POR) Isoforms Play Distinct Roles in Environmental Adaptation in Rice
Source: Rice (N Y). 2017 Jan 11;10:1. doi: 10.1186/s12284-016-0141-2 (PMC5226909; doi:10.1186/s12284-016-0141-2)
Supplement: Additional file 1: Figure S1. — PCR-based confirmation of OsPORA insertion in genomic DNA of three independent OPAO T0 lines. (PDF 136 kb) [file 12284_2016_141_MOESM1_ESM.pdf]

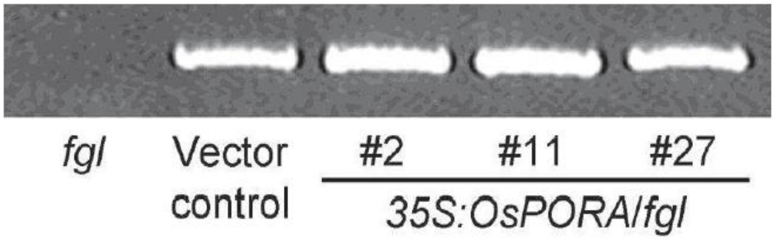

Additional file 1: Figure S1 PCR-based confirmation of *OsPORA* insertion in genomic DNA of three independent OPAO T_0 lines.

Genomic DNA from *fgl* mutant and the *35S:OsPORA* plasmid used for plant transformation were used as negative and positive controls, respectively.
