# Supplementary material for: Two NADPH: Protochlorophyllide Oxidoreductase (POR) Isoforms Play Distinct Roles in Environmental Adaptation in Rice
Source: Rice (N Y). 2017 Jan 11;10:1. doi: 10.1186/s12284-016-0141-2 (PMC5226909; doi:10.1186/s12284-016-0141-2)
Supplement: Additional file 2: Figure S2. — Expression levels of OsPORA in the 2nd leaves of OPAO T1 lines. (PDF 125 kb) [file 12284_2016_141_MOESM2_ESM.pdf]

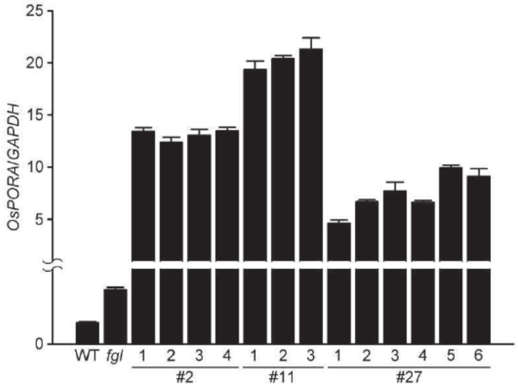

Additional file 2: Figure S2 Expression levels of *OsPORA* in the 2nd leaves of OPAO T_1 lines. The plants were grown for 2 months in a paddy field.
