# Supplementary material for: Two NADPH: Protochlorophyllide Oxidoreductase (POR) Isoforms Play Distinct Roles in Environmental Adaptation in Rice
Source: Rice (N Y). 2017 Jan 11;10:1. doi: 10.1186/s12284-016-0141-2 (PMC5226909; doi:10.1186/s12284-016-0141-2)
Supplement: Additional file 3: Figure S3. — Phenotypes of WT, fgl mutant, and OPAO homozygous T2 line #11 in the paddy field. (PDF 573 kb) [file 12284_2016_141_MOESM3_ESM.pdf]

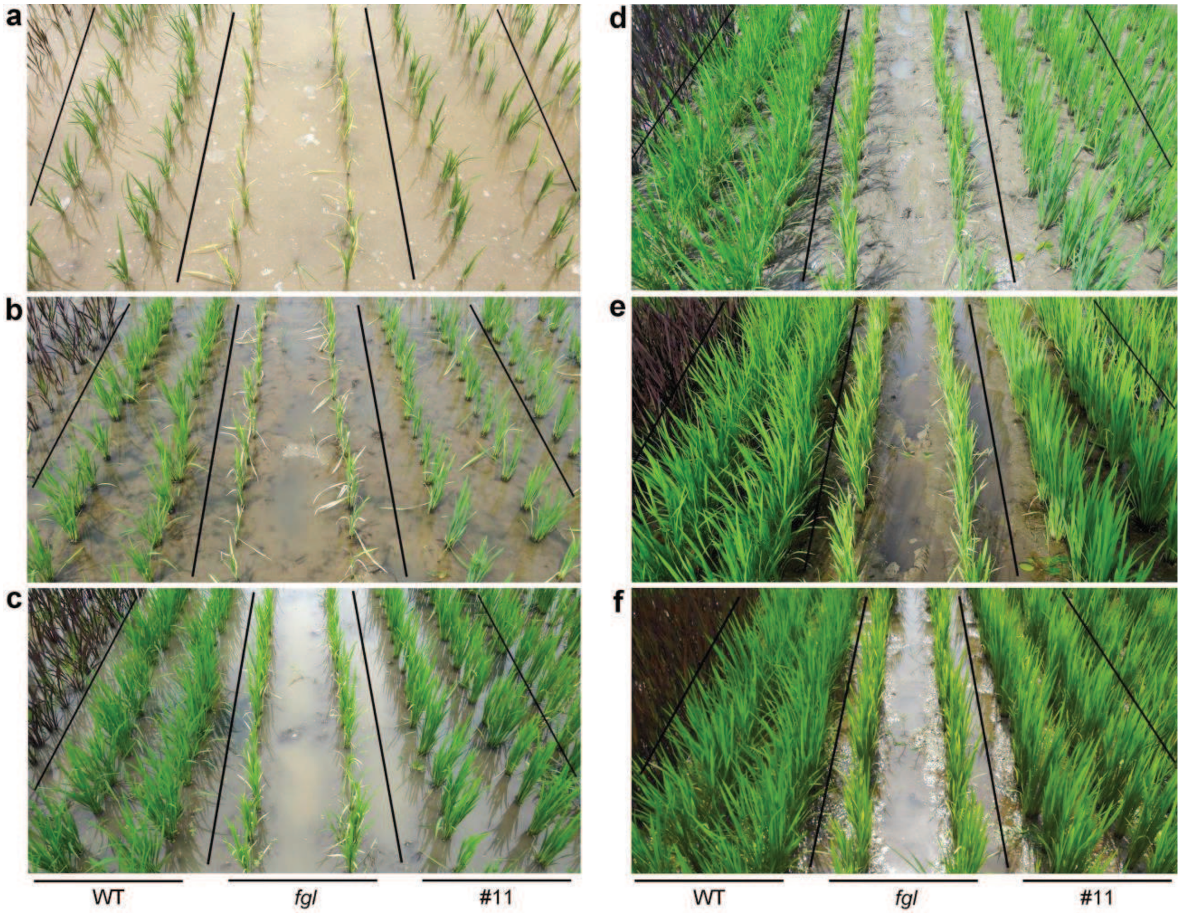

Additional file 3: Figure S3 Phenotypes of WT, *fgl* mutant, and OPAO homozygous T_2 line #11 in the paddy field.

a-f Each panel shows plants at 60 (**a**), 65 (**b**), 70 (**c**), 75 (**d**), 80 (**e**), and 85 days after sowing (**f**).
