# Supplementary material for: Two NADPH: Protochlorophyllide Oxidoreductase (POR) Isoforms Play Distinct Roles in Environmental Adaptation in Rice
Source: Rice (N Y). 2017 Jan 11;10:1. doi: 10.1186/s12284-016-0141-2 (PMC5226909; doi:10.1186/s12284-016-0141-2)
Supplement: Additional file 4: Figure S4. — Total Pchlide and photoactive Pchlide levels determined by UV/VIS spectrophotometry. (PDF 175 kb) [file 12284_2016_141_MOESM4_ESM.pdf]

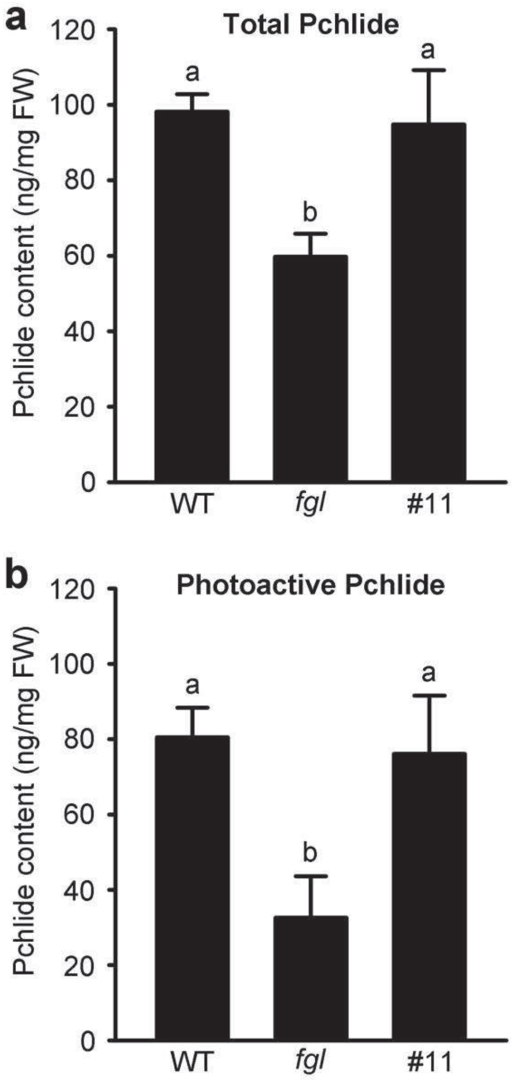

Additional file 4: Figure S4 Total Pchlide and photoactive Pchlide levels determined by UV/VIS spectrophotometry.

a Total Pchlide was measured in aqueous samples from 10-day-old seedlings grown in the dark. **b** Photoactive Pchlide levels were determined based on the difference between Total Pchlide and non-photoactive Pchlide levels. Averages and standard deviations were obtained from 10 independent biological replicates. The experiments were conducted twice and produced similar results. Different letters above the bars indicate statistically significant differences by Duncan's test ($P < 0.01$).
