# Supplementary material for: Two NADPH: Protochlorophyllide Oxidoreductase (POR) Isoforms Play Distinct Roles in Environmental Adaptation in Rice
Source: Rice (N Y). 2017 Jan 11;10:1. doi: 10.1186/s12284-016-0141-2 (PMC5226909; doi:10.1186/s12284-016-0141-2)
Supplement: Additional file 5: Figure S5. — Phenotypic characterization of OPAO line #11 under SD conditions. (PDF 339 kb) [file 12284_2016_141_MOESM5_ESM.pdf]

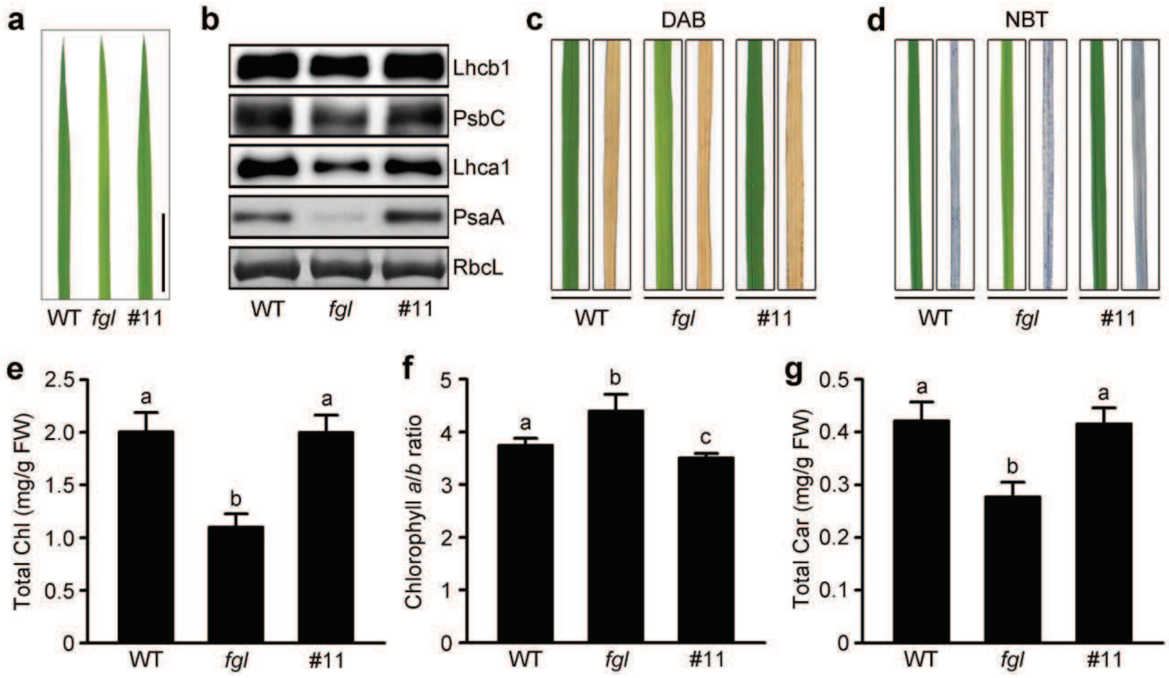

Additional file 5: Figure S5 Phenotypic characterization of OPAO line #11 under SD conditions.

a Leaf phenotypes of WT, *fgl* mutant, and OPAO line #11. The third leaf blades of plants grown for 3 weeks were observed. Scale bar = 2 cm (**a**). **b** Levels of photosystem proteins in leaf blades. The proteins were visualized using specific antibodies (Lhcb1, PsbC, Lhca1, and PsaA) or Coomassie Blue staining (RbcL) (**b**). **c-d** Confirmation of ROS accumulation. H_2O_2 and O_2^- in leaves were detected by DAB (**c**) and NBT (**d**) staining, respectively. **e-g** Determination of total Chl contents (**e**), Chl *a*/*b* ratios (**f**), and total Car contents (**g**) in leaf blades. The data were obtained from 10 independent samples, and different letters above the bars indicate significantly different values (Duncan's test, $P < 0.01$) (**e-g**). All experiments were repeated at least twice and produced similar results.
