# Supplementary material for: Two NADPH: Protochlorophyllide Oxidoreductase (POR) Isoforms Play Distinct Roles in Environmental Adaptation in Rice
Source: Rice (N Y). 2017 Jan 11;10:1. doi: 10.1186/s12284-016-0141-2 (PMC5226909; doi:10.1186/s12284-016-0141-2)
Supplement: Additional file 6: Table S1. — Primers used in this study. (PDF 125 kb) [file 12284_2016_141_MOESM6_ESM.pdf]

Additional file 6: Table S1. Primers used in this study

Name	Forward (5' → 3')	Reverse (5' → 3')
OsPORA cDNA	ATGGCTCTCCAAGTTCAGGC	TCAGACGAGGCCGACGAGCT
OsPORA insertion	CTATCCTTCGCAAGACCCTT	CGGGTAGTCGGATTTCTTGAGG
OsPORA qPCR	ATGGCTCTCCAAGTTCAGG	CTTCTGGCTCACGCTAAGGAAC
GAPDH qPCR	AAGCCAGCATCCTATGATCAGATT	CGTAACCCAGAATACCCTTGAGTTT
CHLH qPCR	AACTGGATGAGCCAGAAGAGA	AAATGCAAAAGACTTGCGACT
GSAT qPCR	AGAACAAAGGGCAGATTGCT	CCAAAAGTGCACCGTCTTGT
DVR qPCR	GATCCATACCCGATCGACAT	CGAGAGACATCCGGTAGAGC
Lhcb1 qPCR	CCATGTTCTCCATGTTCGGCTTCT	TAGGCCCAGGCGTTGTTGTTGA
Lhcb4 qPCR	TACCTGCAGTTCGAGCTGGAC	AGGCCGAACACCTCGGTGTA
